# Understanding Dermatologic Concerns Among Persons Experiencing Homelessness: A Scoping Review and Discussion for Improved Delivery of Care

**DOI:** 10.1177/12034754211004558

**Published:** 2021-04-04

**Authors:** Merna Adly, Taylor Evart Woo, Danya Traboulsi, David Klassen, Jori Hardin

**Affiliations:** 1 70401 2129 University of Calgary, Cumming School of Medicine, Calgary, AB, Canada; 2 Department of Dermatology, Cumming School of Medicine, Calgary, AB, Canada; 3 Department of Community Health Sciences, Cumming School of Medicine, Calgary, AB, Canada

**Keywords:** dermatology, homelessness, infestations, infections, skin cancer, review

## Abstract

There is a paucity of information surrounding dermatologic care for persons experiencing homelessness (PEH). This scoping review aims to map existing literature and provide a summary of the most common cutaneous manifestations among PEH, risk factors for dermatologic disease, describe any reported interventions, as well as identify research gaps for future studies. Search strategies developed for MEDLINE and hand searching yielded 486 articles. Out of the 486 articles screened, 93 articles met the inclusion criteria. The majority were cohort studies, cross-sectional studies, and case-control studies concentrated in North America and Europe. Excluding the pediatric population, the prevalence of dermatologic conditions ranged from 16.6% to 53.5%. Common skin conditions described in PEH were: acne, psoriasis, seborrheic dermatitis, atopic dermatitis, and lichen simplex chronicus. There were no studies comparing the extent or severity of these cutaneous diseases in PEH and the general population. PEH have a higher prevalence of skin infections and non-melanoma skin cancers. This scoping review has direct implications on public health interventions for PEH and highlights the need for evidence-based interventions to provide optimum and safe dermatologic healthcare for PEH. We propose several recommendations for improved care delivery, including addressing upstream factors and comorbidities impacting skin health, providing trauma informed care, reducing barriers to care, preventing and managing skin conditions, as well as including PEH in the planning and implementation of any proposed intervention.

## Introduction

The Canadian Observatory on Homelessness (2012) describes homelessness as “the situation of an individual, family, or community without stable, permanent, appropriate housing or the immediate prospect, means and ability of acquiring it.”^1^ Homelessness encompasses a range of housing and shelter circumstances, including unsheltered, emergency sheltered, provisionally accommodated, and those at risk of homelessness.^1^ At risk individuals are not experiencing homelessness, but their current economic and/or housing situation is precarious or does not meet public health and safety standards.^1^ According to the most recent reports, 35 000 Canadians are homeless on a given night and at least 2 35 000 Canadians experience homelessness in a year.^2^ Of these individuals, 27.3% were women, 18.7% were youth, and 6% were recent immigrants or migrants.^2^ Indigenous individuals, refugees and other newly arrived immigrants in Canada, individuals living with disability, members of lesbian, gay, bisexual, trans, queer, intersex, asexual, and 2-spirit(LGBTQIA2S) communities, people with mental health and substance use problems, and people exiting institutions such as correctional facilities or foster care are overrepresented among those experiencing homelessness.^3^ Persons experiencing homelessness face significantly higher rates of morbidity, mortality, and hospitalization rates due to extreme poverty, congested living conditions in shelters, harsh living environment predisposing them to the extremes of weather, challenges with adherence to medical recommendations, competing needs for sustenance, lack of social support, and discrimination and stigmatization in healthcare settings.^4^ Previous research showed that PEH have higher rates of dermatologic conditions.^4^ We conducted a scoping review to summarize the available literature on the epidemiology and prevalence of dermatologic conditions among PEH, describe the risk factors for and complications of these dermatologic conditions, describe any reported interventions and suggest healthcare delivery improvements for PEH.

## Material and Methods

### Overview

Scoping reviews aim to capture and map information on a broad topic to identify general knowledge gaps and provide conceptual clarity from a wide range of research material.^5-7^ We utilized the general six step approach described by Arskey and O’Malley for conducting scoping reviews,^5-7^ surrounding our research questions of “What is the prevalence of dermatologic conditions amongst PEH?”, “What are the common dermatologic conditions amongst PEH?”, “What are risk factors for dermatologic conditions amongst PEH?”, and “What are described interventions for dermatologic care provision?”

### Information Source and Search Strategy

To balance breadth of review and feasibility, a comprehensive literature search was performed of the MEDLINE database, up to and including May, 15, 2020 using keyword and MeSH search terms relating to:

(Homeless OR Homeless Persons OR Homeless Youth OR runaway* OR Fixed address OR roofless OR street people OR underhouse* OR street youth OR street involved OR sleeping rough OR unstable hous* OR housing instability OR precarious hous* OR vulnerable hous* OR vulnerable populations) AND (skin OR derm* OR skin infection OR skin disease OR wound OR skin cancer OR tinea OR cutaneous OR hair OR nails OR pediculosis OR scabies OR cellulitis).

Articles were subsequently restricted to English language.

### Eligibility Criteria

All primary original research articles, review articles, and case studies that reported on dermatologic condition in PEH were included in this scoping review. Inclusion criteria were: (a) inclusion of patients experiencing homelessness, a broader definition was utilized including both sheltered and unsheltered individuals, “street people,” and those transiently living; (b) study findings or outcomes focused either directly on dermatologic disease or in association to any systemic conditions with cutaneous manifestations or conditions that may impact skin, hair, or nails. Articles that did not present any data on dermatologic conditions or cutaneous manifestations of systemic disease among PEH were excluded. Commentaries, viewpoints, and conference abstracts were excluded.

### Article Selection and Data Extraction

The MEDLINE search results yielded 467 articles. Two reviewers (M.A., T.W.) completed the initial review of abstracts and titles. Discordance among raters resulted in a review by a third rater (J.H.). If it was unclear whether an article met the inclusion criteria, the full text article was retrieved to determine eligibility. Upon identification of articles that met the inclusion criteria, M.A. extracted the data. A standardized data extraction form was developed to collect the following from primary research articles, including general demographic characteristics, setting, study design, dermatologic condition assessment method, pertinent dermatologic findings, and quality score (Supplemental Table S1). A standardized data extraction form was also developed to collect the following from case studies, including demographic characteristics, clinical findings, diagnosis, outcomes and significance of study (Supplemental Table S2).

### Assessment of Study Quality and Data Synthesis

Study quality was assessed by M.A. and T.W. using the Oxford Centre for Evidence-Based Medicine Levels of Evidence Scale,^8^ which provides as assessment of study quality on numeric scale from 1 (best quality) to 5 (lowest quality), and an overall level of evidence for the studies included from A (highest level of evidence) to D (lowest level of evidence). Discrepancies in quality score assessment for each study were resolved by consensus. After data extraction from eligible studies, data were qualitatively synthesized and reported descriptively. The article was prepared using the PRISMA-Scoping Review checklist as a guide.^9^

## Results

### Study Selection and Characteristics

Study selection is depicted in the flow diagram (Supplemental Figure S1). Initially, 467 articles were identified from the database. After initial screening of abstracts and titles according to guidelines above, 132 articles remained. Of these articles, a further 58 articles were further excluded because they did not address PEH or did not provide dermatologic health outcome data in the full-text according to pre-specified inclusion/exclusion criteria. The reference lists of included studies in the review were hand searched until saturation for identification of any additional article. A further 19 articles were identified through hand searching. In total, 93 articles were included in qualitative synthesis. The breadth of the review questions and heterogeneity of the study necessitated a narrative synthesis of the data. Meta-analysis was not performed within this scoping review.

### Study Design and Dermatologic Condition Assessment

A total of 71 original articles,^10-80^ 9 case studies,^81-89^ 9 review articles,^90-98^ and 4 systematic reviews^99-102^ met the inclusion criteria and were included for analysis. Most original studies were observational and descriptive, including cohort, cross-sectional, and case control studies (Supplemental Table S1). There was one randomized controlled trial,^61^ and few qualitative studies identified in the scoping search.^26,77^ The methodology of most primary research articles involved chart review analysis, questionnaires and surveys, and lab culture results (Supplemental Table S1). Most studies involved assessment of dermatologic conditions through physical exams conducted by clinicians. No study compared dermatologic outcomes in those who remained homeless compared to those who were able to obtain housing.

### Methodological Quality

The methodological quality in the majority of original primary studies was generally moderate with a median score of 3 (range 1-4). There was heterogeneity among the studies with regard to clinical factors, methodological information, and inclusion criteria. There were few studies that had low internal and external validity and appeared to be insufficiently powered to detect clinically meaningful difference. The overall quality of evidence was level B on the Oxford Centre for Evidence-Based Medicine Levels of Evidence Scale^8^ (Supplemental Table S1).

### Setting

The 71 extracted primary original research articles and 9 case studies were conducted in a broad range of countries and included shelter residents and patients from outreach facilities and dedicated PEH clinics (Supplemental Table S1).

### Sample

The sample size of the primary literature ranged from 40 to 18 864 participants (Supplemental Table S1). Most studies utilized reporting of mean age, age range, or median for their study cohort. The mean age ranges from all the adult studies ranged from 32 to 57 (Supplemental Table S1). Five studies involved the pediatric population; 3 studies were describing ‘street children’,^14,26,51^ and 2 studies were describing sheltered PEH.^34,68^ With the exception of 4 articles, the majority of patients were males.^34,43,49,57^ The definition of homelessness varied across studies, and occasionally it was not adequately defined or described.

## Dermatologic Conditions in Adult Population

Dermatologic conditions were one of the major physical health concerns among PEH.^10,13,15,49,54,57,80^ According to all extracted studies, the prevalence of skin conditions ranged from 16.6% to 53.5%.^10,11,15,25,57,64,80^ Several studies described statistically significant higher prevalence of dermatologic conditions among PEH compared to the general population.^11,19,25,41^ The prevalence of common skin conditions described among PEH such as acne,^32,37,64^ psoriasis,^37,64^ seborrheic dermatitis,^32,37^ atopic dermatitis,^37,64^ lichen simplex chronicus,^64^ pruritus,^25^ and skin trauma/injuries^15,32,55,64^ is reported in Table 1. The average scores for dermatology life quality index (DLQI) for acne, psoriasis, eczema and skin cancer were generally higher among PEH when compared to the values described among non-homeless populations.^76^ Interestingly, higher DLQI was associated with being a non-white patient, poorer skin health, and a rash or infectious diagnosis.^76^ A study describing treatment disparities for common skin conditions among PEH compared to the non-homeless population demonstrated a pattern of less diagnostic inquiry, less aggressive intervention and fewer recommendation for follow-up for common skin conditions, despite the same dermatologists treating the same groups.^75^

**Table 1:** The Prevalence Ranges of Dermatologic Conditions Described Among Persons Experiencing Homelessness (PEH).

Dermatologic condition	Prevalence range
Acne	3.1% to 19.8%^32,37,64^
Psoriasis	4.3% to 7.8%^37,64^
Seborrheic dermatitis	1.6% to 13.3%^32,37^
Atopic dermatitis	12.5% to 15.9%^37,64^
Lichen simplex chronicus	5.6%^64^
Pruritus	36.3%^25^
Skin trauma/injuries	13% to 19.7%^15,32,55,64^
Body lice	0.15% to 30%^25,46,52,58-60,64^
Head lice	4.5 %to 4.9%^58,59^
Scabies	0.4%-6.5^25,28,52,64,65^
Pubic lice	3.2%^59^
Tinea pedis	3.1% to 38%^25,37,64^
Tinea cruris	1.6%^37^
Onychomycosis	1.6% to 5.2%^25,37^
Cellulitis	11.1% to 14%^64,66^
Folliculitis	4.8% to 5.1%^25,65^
Impetigo	2.4%^25^
Immersion foot	5% to 17%^69,80^
Pitted keratolysis	20.4%^32^
Corns and Calluses	7.7% to 57%^99^
Foot nail pathologies	15.0% to 65%^99^
Foot infections	3.2% to 38%^99^
Frost bite	2%^69^
Chlamydia	Adults: 6.4% to 6.7%^101^ Pediatrics: 18.3% to 40.9%^102^
Gonorrhea	Adults: 0.3% to 3.2%^101^ Pediatrics: 0.4% to 24.9%^100^
Herpes	Pediatrics: 1.1% to 11.8%^100^
Genital warts	Pediatrics: 3.5%^100^
Syphilis	Adult: 1.1%^101^ Pediatrics: 0.2% to 3.5%^100^
Human papilloma virus (HPV)	Pediatrics: 1.3%^100^
Trichomonas vaginalis	Pediatrics: 0.7%^100^

## Infestations and Infections

### Skin Infestations Among PEH

Persons experiencing homelessness have a higher prevalence of ectoparasitic infections due to poor living situations, overcrowding, lack of access to regular bathing facilities, poor hygiene, and disease unrecognition.^25,28,92^ Several studies describe higher prevalence of infestations, including body lice,^25,28,46,52,58-60,64^ head lice,^58,59^ scabies,^25,28,52,64,65^ and pubic lice.^59^ The prevalence of these conditions is described in Table 1. Unsheltered PEH were more frequently affected with pediculosis corporis.^52,58^ Other statistically significant risk factors for pediculosis corporis included male sex, African-American ethnicity, substance usage, lack of access to bathing facilities, and previous history of pubic lice.^52,58,59^ Statistically significant risk factors for scabies included being a woman, living in an abandoned place, and not possessing a sleeping bag.^52^

Ectoparasitic infestations, namely body lice, are potential vectors for several infectious agents, particularly Bartonella quintana (causing trench fever),^28,58,60,62,94^ *Rickettsia prowazekii* (causing epidemic typhus),^28^ and *Borrelia recurrentis* (causing relapsing fever).^28,60^ Positive serology with *Bartonella quintana* has been documented extensively in PEH, however, there is less data on the prevalence of *Rickettsia* species and *Borrelia* species. Ectoparasite infestations may be associated with systemic complications, with several cases of iron-deficiency anemia being associated with heavy and prolonged lice infestations among PEH.^89^

There is limited data describing the impact of medical interventions on controlling louse infestations among PEH. A randomized controlled trial (RCT) among sheltered PEH in France showed that individuals who received permethrin-impregnated underwear were freer of lice on day 14 compared with placebo; however, on day 45, the results were not sustained and there was higher prevalence of permethrin resistance.^61^ In another study, it was found that the prevalence of body lice infested individuals fell from 84.9% to 18.5% over a 2 week period after administration of 3 doses of oral ivermectin (12 mg each) given at days 0, 7, and 14.^73^ Although this effect was not sustained at day 45, it suggests that ivermectin may play a novel role in control of body louse infestations in humans.^73^

### Skin Infections Among PEH

#### Bacterial infections

Several studies have examined the increased prevalence of bacterial-related infections, including abscesses and cellulitis, folliculitis, and impetigo among PEH (Table 1).^25,65^ Bacterial skin infections were one of the most common reasons for emergency department (ED) visits and hospital admission among PEH, particularly in people who inject drugs (PWID).^12,18,22,30,38,67^ Risk factors for cutaneous injection related infection (CIRI) among PEH who use drugs included being female, needle/syringe re-usage, usage of borrowed needles, challenges with hygiene, requiring help injecting, daily cocaine injection, repetitive skin breakdown, and skin breach in order to achieve drug entry.^16,17,30,38^ Qualitative accounts of PEH and PWID demonstrated engagement with the medical system was a last resort, with admission to hospital in critical or a “near death” condition.^77^ Postulated barriers to health access may include feeling marginalized, judged, medicalized in relation to drug use, being stigmatized due to wound odor or appearance of homelessness, as well as the unequal power relations with medical professionals.^77^

#### Methicillin resistant Staphylococcus aureus (MRSA) infections

Homelessness was associated with increased prevalence of methicillin resistant *Staphylococcus Aureus* (MRSA) colonization and skin and soft tissue infections.^21,23,24,27,36,39,43,44,63^ Risk factors associated with MRSA colonization or infection included: a history of incarceration or alcohol use disorder; frequent emergency department visits; previous history of MRSA infection; previous usage of antibiotics in the last 6 months; history of multiple abscesses; sleeping at more than one location in the last week; living in a communal setting; using public shower facilities; and skin manipulation.^21,23,24,27,36,39,44,63^

#### Group A Streptococcus (GAS) infections

Several studies showed higher incidence of invasive Group A Streptococcus (iGAS) outbreaks and infections manifesting as cellulitis and necrotizing fasciitis with associated higher mortality rates among PEH.^42,47,48,50,53,70,78^ Compared to the general population, PEH were 53.3 to 100 times more likely to have iGAS infection.^47,70,78^ Factors associated with Group A Streptococcus (GAS) colonization and infection included: sleeping outside; poor hygiene; sharing personal items with others; skin breakdown; previous skin infection; alcohol use disorder; as well as being male and of younger age.^42,48,78^ A study suggested mass administration of a single dose of one gram of Azithromycin led to reduction of iGAS incidence from 1.5 to 0.2 cases per 1000 PEH.^50^ Another study suggested that prevention of GAS infections may be achieved through better provision of wound care and hygiene support.^47^

#### Fungal infections

The prevalence ranges of fungal infections were found to be higher among PEH and is summarized in Table 1.

#### Cutaneous diphtheria

Few studies described higher incidence of *cutaneous diphtheriae* which is described as chronic, nonhealing ulcers due to physical trauma, and/or underlying dermatoses that are superinfected with *Corynebacterium diphtheriae*.^56,71,72,88^ In Canada, cutaneous *diphtheriae* outbreaks have been described in Vancouver Downtown East Side (VDES), an inner-city community with high rates of homelessness, drug use, and HIV infection.^71,72^ Overcrowding, poor hygiene and personal proximity were associated with *Corynebacterium diphtheriae* colonization.^56^

#### Sexually transmitted infections (STIs)

Adult STI prevalence range was 2.1% to 6.7%; the prevalence ranges of chlamydia, gonorrhea, and syphilis is reported in Table 1.^101^ Higher STI prevalence among PEH was associated with intimate partner violence, injection and noninjection substance use, incarceration, and homelessness severity (measured by the number of episodes of homelessness, number of years homeless, and prior shelter stays).^101^

## Skin Cancer

There is a higher prevalence of malignant/pre-malignant growth among PEH compared to general population (25%, 6.1, *P* < .0001),^24^ likely due to chronic sun exposure, limited education regarding skin cancer risk reduction, lack of sun protective resources, and reduced access to healthcare.^29,35,37^The prevalence of non-melanoma skin cancer rates including pre-malignant lesions in PEH ranged from 10.9% to 25%.^35,37,66,76^ Lower prevalence rates were described in younger populations and may be attributed to less cumulative lifetime sun exposure.^32,65^Studies showed that 71% to 76% of PEH have never had a skin exam^29,40^; 50% to 79% of PEH have never used sunscreen^29,35,40^; 33% never wore a hat during sun exposure^29^; and 45% to 50% sought out shaded areas when possible.^35^ Finally, a majority of participants (88%) reported they would be willing to use sunscreen and sun protective clothing if it was available to them free of cost.^29^

## Foot Conditions

Immersion foot,^69,80^ pitted keratolysis,^32^ corns and calluses,^69,99^ as well as frost bite and foot injuries^69,99^ were common among PEH (Table 1). This is due to prolonged exposure to moisture, long periods of walking and standing, limited access to clean socks and properly fitting footwear, and barriers to hygiene.^64,69,81^ Furthermore, other comorbidities prevalent among PEH such as peripheral neuropathy secondary to alcohol or diabetes, peripheral vascular disease, and usage of vasoconstrictive drugs such as cocaine may predispose individuals to infections and poor wound healing.^64,69,81^ Foot related skin diseases, which include lymphedema, venous stasis disease, and tinea pedis infections are predisposing factors for cellulitis, skin ulcers, gangrene, chronic non-healing wounds, and osteomyelitis.^67^

## Malnutrition

There were 3 cases of pellagra (niacin deficiency) described.^83,86^ None of these individuals had a history of excessive alcohol consumption. There was one case study describing vitamin A deficiency with cutaneous manifestations (follicular keratosis) which resolved with treatment.^82^

## Dermatologic Conditions in Pediatric Population

Several reports described dermatologic conditions in pediatric PEH; 3 studies reported on ‘street children’ in Egypt, Nepal, and Pakistan^14,26,51^ and 2 studies reported on sheltered PEH in the United States.^34,68^ Skin conditions were one of the most prevalent physical concerns (10.0% to 50.9%) among pediatric PEH in Egypt, Nepal, and Pakistan.^14,26,51^ Common conditions included skin infections, head lice, rash, itch, eczema, and scabies.^14,26,51^ In the United States, dermatologic conditions were also one of the major physical concerns among youth experiencing homelessness (prevalence: 31.9%).^34^ Common skin conditions included acne, atopic dermatitis and burns.^34,68^ Skin conditions were the most common reason for healthcare visits among males.^34^ Among youth experiencing homelessness (12, 23 years old), the overall STI prevalence ranged from 6.0% to 32.0%; among girls rates ranged from 16.7% to 46.0% while rates for boys ranged from 9.0% to 13.1%.^100^ The reported ranges of herpes, genital warts, syphilis, human papilloma virus (HPV) and Trichomonas vaginalis among youth is reported in Table 1.^100,102^ Finally, among children experiencing homelessness, the prevalence of child abuse is high (a rate of 8.8 case per 1000 children has been reported).^67^ Cutaneous manifestations of abuse may include multiple injuries in different stages of healing over many body sites; unusual patterns of scaring and bruising; and suspicious pigmentary changes.^67^

## Discussion

Persons experiencing homelessness experience disproportionately higher prevalence of dermatology-related concerns. Higher prevalence rates of ectoparasitic infestations, bacterial and fungal skin infections, cutaneous injection-related infections, and skin cancer were reported. Persons experiencing homelessness face higher skin disease burden due to chronic exposure to extremes of weather, lack of access to adequate clothing, limited access to hygiene measures, congested living conditions, and lack of privacy. Importantly, PEH also face unique challenges in the management of chronic dermatologic conditions due to difficulties with applying large quantities of topical medications, as well as contraindications to commonly used therapies due to comorbid systemic disease such as tuberculosis, HIV, or viral hepatitis.^103^ Furthermore, PEH were less likely to seek medical attention for a variety of reasons including fear of stigmatization or comorbid psychiatric illness. Our extracted literature described several models of outpatient dermatologic care provision, including free clinics dedicated for dermatologic care of PEH at shelters and communities. Practicing in environments that are safe and low barrier for PEH was key in effective dermatologic care provision. Tele-dermatology was also reported as a viable option for care provision for vulnerable populations.^84^ Within a Canadian context, there should also be better lines of communication between dermatologists, other specialists, family physicians, and public health officials to ensure that PEH with complex dermatologic needs receive the right care at the right time.

Limitations of our review include a small number of randomized controlled trials and large scale prospective/retrospective studies; heterogeneity due to inconsistent definition of homelessness; settings with differing levels of access to medical care and resources; differing living situations; variable assessment methods and heterogeneity of clinical characteristics. Given the reported literature likely favors the publication of cases describing dermatologic conditions among PEH, there may be an element of publication bias. Future studies dedicated to the dermatologic health needs of women and children experiencing homelessness are crucial, as most of our extracted studies focused on men. Future research may also focus on the impact of intersectionality on dermatologic health.

## Recommendations

In order to reduce the morbidity and mortality of dermatologic conditions associated with homelessness, it is necessary to address upstream structural determinants and material circumstances such as housing, income assistance, food security, as well as downstream health related consequences of inadequate housing. The following recommendations are intended to provide initial steps for clinicians, as well as public health and policy makers. It will also be crucial for PEH to be involved in the planning and implementation of all levels of intervention.

Recommendation I: Address basic necessities, such as housing, income, and food security to improve dermatologic health outcomes among PEH.

A primary goal should be to end homelessness through permanent supportive housing (PSH) and income assistance programs.^104^ The use of poverty screening tools to identify income insecurity or housing instability, and collaboration with others to assist with identifying income-support resources may positively impact skin health along with assuring food security and basic hygiene tools. Screening questions to identify housing instability may include “What is your housing situation today?”, “In the past 2 months, have you been living in stable housing that you own, rent, or stay in as part of a household?” and “Are you worried or concerned that in the next 2 months you may NOT have stable housing that you own, rent, or stay in as a part of a household?”^103^ When housing instability is identified, clinicians are encouraged to incorporate elements of social determinants of health (SDOH) screening into their practice by asking follow-up questions regarding access to water, sanitation, and hygiene facilities.^103^Recent guidelines provide strong recommendations for clinicians to identify PEH, in order to ensure patient access to housing coordinator or case managers for link to PSH.^105^

Recommendation II: Provision of trauma informed care and culturally safe care, particularly for special populations, including Indigenous populations, immigrants and refugees, and women.

Many PEH feel unsafe disclosing sensitive information to health care providers.^3^ Inequity responsive care, trauma-informed, culturally safe and contextually tailored care may reduce barriers to care by creating safe spaces for disclosure and trusting relationship between physicians and patients.^3^ Trauma informed care is based on 5 main tenants: (1) bearing witness to the patient’s experience of trauma, (2) helping patients feel safe, (3) involving patients in the healing process using informed choice, (4) recognizing and building on patient strengths, (5) incorporating processes that are sensitive to a patient’s culture, ethnicity, personal and social identity, as well as recognizing that certain populations may experience systemic abuse that leads to intergenerational and lateral transmission of both trauma and shame.^3,106^ Thistle & Smylie provide one example of protocols to follow when providing care for Indigenous individuals experiencing homelessness: (1) Situating one’s self by educating oneself about colonial history, taking evidence-based cultural safety training, and introducing oneself as someone who works in Indigenous territory, (2) “Visiting” by ensuring adequate time with each patient and ensuring patients’ comfort, (3) “Hospitality” by including Indigenous-specific features, symbols and artwork , as well as having Indigenous staff, (4) “treating people as you would treat your own relative.”^107^ When interacting with immigrant and refugee populations, it is essential to recognize the impact of discrimination, limited language proficiency, and severed social networks on the population’s sense of belonging and access to medical and social services.^105^ When interacting with women experiencing homelessness, clinicians should focus on patient safety, empowerment among women faced with gender-based violence, and improved access to PSH and resources, including income, childcare, and social support.^105^ Clinicians should also take the time to understand implicit bias and its impact on patient care. Lastly, clinicians should be aware of the impact of intersectionality on dermatologic health outcomes.

Recommendation III: Address comorbid health needs among PEH to improve skin health.

Addressing mental health and addiction may reduce the incidence of skin concerns such as infections, infestations, picking, and challenges with self-care. Current guidelines provide conditional recommendations for clinicians to identify history of mental health concerns or substance use disorder to facilitate connection to local community addiction and mental health programs, case management, or harm reduction interventions.^105^ Harm reduction interventions, including Needle Exchange Programs (NEP) and Supervised Consumption Facilities (SCF), may mitigate unsafe drug use behaviors leading to skin infections through the provision of clean drug supplies.^108,109^ In addition, these interventions may enable access to ancillary health and community services and resources (shelter and food).^110^

Recommendation IV: Management of skin issues (dermatology specific recommendations)

Our scoping review emphasizes the importance of effective preventive approaches for improved cutaneous care delivery for PEH, particularly hygiene optimization,^96^ provision of non-judgemental multi-disciplinary drop-in wound care clinics for PWID,^97,98^ as well as skin cancer screening and education on sun protection.^29,35,37^ Clinicians may address the unique barriers to dermatologic disease management surrounding limited access to privacy, basic hygiene, and cost of medications by developing person-centered care plans. For instance, when treating scabies, oral ivermectin and provision of clean clothing may be more appropriate than the conventional recommendation of topical permethrin, which assumes that patients have privacy and access to water and extra clothing.^103^ When managing skin or soft tissue infections among PEH, clinicians should assess for an underlying disease predisposing to bacterial super-infection, such as louse infestations, dermatophyte infections, venous stasis or injection drug use.^103^To address medication costs, clinicians may need to work with case managers or social workers to improve access to subsidized or free medications as well as ancillary services to offset medication-related expenses. Clinicians may also need to work with non-profit organizations to improve access to hygiene products, sunscreens, and emollients given that majority of PEH lack access to skincare resources.^76^ Moving forward, we need to generate evidence for these suggested interventions, particularly their impact on health outcomes and healthcare utilization costs.

Recommendation V: Re-orientation of curricula to graduate clinicians capable of addressing the needs of vulnerable population.

Teaching clinicians on providing person-centered care plans for PEH may involve providing didactic teaching on the needs of PEH, training on trauma-informed care and implicit bias, as well as providing opportunities for experiential learning in community settings. In these settings, future clinicians may develop an appreciation for the impacts of material circumstances on health, as well as develop the critical lens needed to address health inequities, that is, to act as advocates for change at a system level.

## Conclusion

It is important to focus on skin health among PEH. This review highlights the need for evidence-based interventions to address the dermatologic health needs of this population. We need to work to end homelessness; reduce barriers PEH face in accessing health care including skin care; address common comorbidities among PEH that impact their skin health; prevent and manage dermatologic conditions prevalent among this population.

## Supplemental Material

Supplementary Material 1 - Supplemental material for Understanding Dermatologic Concerns Among Persons Experiencing Homelessness: A Scoping Review and Discussion for Improved Delivery of Care Click here for additional data file. Supplemental material, Supplementary Material 1, for Understanding Dermatologic Concerns Among Persons Experiencing Homelessness: A Scoping Review and Discussion for Improved Delivery of Care by Merna Adly, Taylor Evart Woo, Danya Traboulsi, David Klassen and Jori Hardin in Journal of Cutaneous Medicine and Surgery
