# Supplementary material for: Understanding Dermatologic Concerns Among Persons Experiencing Homelessness: A Scoping Review and Discussion for Improved Delivery of Care
Source: J Cutan Med Surg. 2021 Apr 4;25(6):616–26. doi: 10.1177/12034754211004558 (PMC8640276; doi:10.1177/12034754211004558)

Supplemental Material

Understanding Dermatologic Concerns Among Persons Experiencing Homelessness: A Scoping Review and Discussion for Improved Delivery of Care

[1]Merna Adly, BSc

[2] Taylor Evart Woo, BSc, MD, MSc

[2]Danya Traboulsi BSc, MD, FRCPC

[3]David Klassen, BA, MD, CFPC

[2]Jori Hardin BSc, MSc, MD, FRCPC

[1]University of Calgary, Cumming School of Medicine, Calgary, AB, Canada

[2]Department of Dermatology, Cumming School of Medicine, Calgary, AB, Canada

[3]Department of Community Health Sciences, Cumming School of Medicine, Calgary, AB, Canada

Institution Address and Corresponding Author:

Jori Hardin

Department of Dermatology, University of Calgary

1820 Richmond Road SW

Calgary, Alberta, T2T 5C7

Table S1. Characteristics of included original studies, including demographic information, country, setting, assessment/data collection method, pertinent dermatologic findings and Oxford Centre for Evidence Medicine study quality assessment. Quality level was determined on scale from 1 to 5, with level 1 corresponding to highest quality.

First Author Last Name (year)	Country	Sample Size (% males); % PEH	Mean age or Age range or Median age (years)	Study Design	Setting	Assessment method / Data collection	Pertinent Findings	Study Quality Level (1-5)
Vredevoe. DL (1992)[10]	United States	1252 (91.4%); 100%	Age range: 18-60+	Cohort	Shelter for PEH	Medical records	Majority of health concerns were respiratory (46%), followed by skin (34%), and injuries (21%). Skin concerns were mainly of infectious etiology.	3
Gellberg, L (1990) [11]	United States	464 (56%); 46%	Mean age: 32	Case-control	Shelter for PEH	Medical records and surveys	Compared to the housed poor, PEH were more likely to have dermatological problems; (32% vs 21%).	4
Morris, W (1989)[12]	United States	4243 (NS); 5.3%	Mean age: 38	Cohort	Hospital	Medical records	The most common diagnostic category among PEH discharges was diseases of the skin, subcutaneous tissue and breast (SSTB), constituting 21.2% compared to 8.7% of the discharge diagnoses for housed patients. The main diagnosis under SSTB was cellulitis.	3
Shanks, NJ (1988)[13]	United Kingdom	2500 (96%); 100%	Age range: 15-65+	Cohort	Hostels and day program s	Statistical records	Statistically significant higher consultation rates for psychiatric and dermatologic conditions were reported amongst PEH when compared to the consultation rates reported by the UK Royal College of General practitioners age/sex matched consultation rates.	3
Thapa, K (2009)[14]	Nepal	48 (95.8%); 100%	Age range: 11- 15	Cross-sectional	'Street children'	Interviews and Medical Records	The most common medical concern among the children was head lice infestation (81.7%), followed by itch (51.6%).	3

Van Laere, I (2009)[80]	Netherlands	629 (83%);100 %	Mean age: 45	Cross-sectional	Shelter for PEH	Medical records	The primary physical concern was dermatologic conditions (37%),including immersion foot (17%); skin injuries and infections (13%); erysipelas (12%); and chronic ulcers (4%).	3
Lloyd-Smith, E (2009)[16]	Canada	1080 (71%); 54%	Median age: 38.4	Cohort	SIF	Medical records	The incidence of CIRI was 22.0 per 100 person-years. Factors such as being female, unstably housed, and daily heroin injection were independently associated with receiving CIRI care at the SIF.	3
Beijer, U (2009)[18]	Sweden	PEH: 1704 (80%); 100% Control Group: 5000 (75%); 0%	Mean age for men: 45 Mean age for women: 38	Cohort	Social Welfare Office	Medical records	Both men and women had increased risk of hospitalization due to skin conditions when compared to the control group, with especially higher rates amongst women. Higher rates among women may be attributed to increased physical exposure leading to trauma, difficulties addressing hygiene, and higher rates of amphetamine and heroin usage.	3
Lloyd-Smith, E (2008) [17]	Canada	1065 (71%); NS	NS	Cohort	SIF	Medical records	In the first visit to SIF, the incidence of CIRI was 10%. Female sex, unstable housing, usage of borrowed needles, daily cocaine injection, and requiring help injecting was associated with an increased risk of CIRI.	3
Goldstein, G (2008)[20]	United States	2978 (NS); 100%	NS	Cohort	Community facilities, hospitals, outpatient clinics, prisons, and veterans centers	Interviews and medical records	The most common medical problems amongst veterans experiencing homelessness were dental; orthopedic ; generalized illness; hepatic disorders; and cardiopulmonary disease. Skin conditions were not in the top four medical categories.	3
Skiest, DJ (2006)[21]	United States	190 (64%); 18%	Median age: 43	Cohort	Hospital	Interviews, lab investigations	Patients with MRSA infections were more likely to present with SSTI compared with MSSA patients; (69% vs. 45%, p=0.0012). Univariate analysis showed that African American race, previous MRSA infection, antibiotic usage in the last 6 months, homelessness, incarceration, and alcohol	3

						, and medical records	use disorder were associated with MRSA infection. Multivariate analysis showed that antibiotic usage in the last six months, homelessness, and African American race were associated with MRSA infection.	
O'Toole, TP (2006)[22]	United States	83 (68.7%); 77.1%	Median age: 41.2	Cross-sectional	Substance use program at a hospital	Medical records	The second most common admitting diagnosis amongst PEH was deep tissue abscess / nonhealing abscess or ulcers (18.1%).	3
Gilbert M (2006)[23]	Canada	40 (68%); NS	Median age: 37	Cross-sectional	Shelter for PEH; halfway or transition houses; detoxification centre; jails and other group living facilities	Interviews, lab investigations, and medical records	In MRSA (USA300 strain) outbreak, the clinical presentation was mainly SSTI, including abscess, infected wounds and ulcers, and cellulitis. 70% of individuals in the outbreak had a history of illicit drug use, homelessness, and/or incarceration.	3
Badiaga, S (2005)[25]	France	498 (93%); 100%	Mean Age: 41	Case-control	Shelter for PEH	Medical records	Compared to the control population, PEH have a significantly higher prevalence of skin disease (38% vs 0.5%; p<0.0001); body lice infestation (19.1% vs. 0%; p< 0.0001); scabies (3.8% vs 0%; p<0.0001; folliculitis (4.8% vs 1.5%, p<0.0001); tinea pedis (3.2% vs. 0.5%; p=0.02%); and impetigo (2.4% vs 0%; p<0.0001).	4
Ali, M (2005)[26]	Pakistan	40 (80%); 100%	Age range: 8-14	Cross-sectional/ Qualitative study.	'Street children'	Interviews and medical records	Youth experiencing homelessness are susceptible to injuries, skin and respiratory infections, with 10% having issues like scabies and dermatitis.	3

Frazee, BW (2005)[27]	United States	137(93); 18%	Age range: 18- 60+	Cohort	ED	Lab investigations, medical records, and surveys	Homelessness, frequent ED visits, and history of multiple abscesses were associated with MRSA colonization. White race and furuncle were associated with both MRSA colonization and infection.	3
Brouqui, P (2005)[28]	France	930 (96%); 100%	Mean age: 43	Case-control	Shelter for PEH	Interviews, lab investigations, and medical records	The prevalence of lice and scabies was 22% and 3% respectively. The prevalence of IgG antibodies of the following louse borne blood infections in PEH vs. control population was: (1) *Bartonella quintana (7*.53% vs. 0.6%, p<0.001) ; (2) *Rickettsia prowazekii* (0. 75% vs. 0%, p<0.001); and (3) *Borrelia recurrentis* (1.61% vs. 0%, p<0.001). 72% of participants with *Bartonella quintanta* bacteremia were treated effectively, however, *Bartonella quintana* remained endemic due to louse re-infestations.	4
Chau, S (2002)[29]	United States	221 (54%); 100%	Mean age: 46.7	Cohort	Shelter for PEH, meal programs, and day centre	Medical records and surveys	76% of PEH never had a skin exam; 75% never used sunscreen; and 37% never wore a hat during sun exposure.	3
Palepu, A (2001)[30]	Canada	440 (67%); 63%	NS	Cohort	ED	Medical records and surveys	The most common reasons for ED visit among PWID were SSTI (cellulitis and skin abscess) as well as problems related directly to drug usage (intoxication, overdose, drug withdrawal). Frequent ED usage was associated with unstable housing, HIV infection, more than four daily injections, and cocaine usage.	3
Gelberg, L (2000)[31]	United States	363(80.2%); 100%	Mean age: 38.2	Cohort	Temporary shelters for PEH	Medical records	Skin/leg/foot problems was the second most prevalent category of conditions (prevalence: 36%). However, this category had the lowest health care utilization rates. With the provision of regular source of care through a community clinic, skin/leg/foot problems improved over time.	3

Stratigos, AJ (1999)[32]	United States	142(100%); 100%	Mean age: 38.9	Cross-sectional	Clinics dedicated to PEH	Medical records and surveys	The most prevalent skin disease was tinea pedis (38%); pitted keratolysis of the feet (20.4%); traumatic injuries (19.7%); acne vulgaris (18.3%); onychomycosis (15.4%); seborrheic dermatitis (13.3%); calluses (7.7%). No skin cancer was diagnosed.	3
Gilbert, M (2007)[24]	Canada	271(73.6%) ; 41.3%	Mean age: 37.0	Cross-sectional	Shelters for PEH ;halfway or transition houses; detoxification centre; jails and group living facilities	Lab investigations and surveys	5.5% of participants tested positive for MRSA (USA300 strain). MRSA (USA300 strain) cases were more likely to report manipulation of skin infection, drug use between sex trader workers and clients and with casual sex partners. Data from this study support the need for public health measures to promote appropriate care of skin infections and to caution against usage of expired antibiotics prescription as this may promote antibiotic resistance in this population.	3
Salit, S (1998)[33]	United States	18864 (81.7%); 100%	Age range: 18-64+	Case-control	Hospital	Medical records	Amongst PEH, 51.5% of hospital admission were for substance use or mental illness; 19.7% of admissions were related to trauma, respiratory disorders, skin disorders and infectious disorders, many of which are preventable medical conditions. Compared to control, PEH stayed longer per admission after adjusting for substance use, mental illness and other demographics.	4
Evans, YN (2014)[34]	United States	744(46.8%) ; 100%	Mean age: 18.8	Cohort	Clinics dedicated to PEH	Medical records	Amongst youth experiencing homelessness, common reasons for health care utilization included screening/treatment of STIs (14.3%); physical exam for housing (13.7%); dermatologic exam (13.5%). The most common reason for visits for males were dermatologic conditions (16.4%).	3
Wilde, M (2013)[35]	United States	In **2011**: 29 (NS);100%	NS	Cohort	Clinics dedicated to PEH	Medical records	In 2011, 4 out of 29 patients had suspicious lesions biopsied resulting in a diagnosis of 1 BCC and 1 SCC. In 2012, 10 out of 33 patients had 17 suspicious lesions biopsied resulting in a diagnosis of six BCC and five SCC. Sunscreen was never used by 52% and 30% of population in 2011 and 2012	3

		In **2012**: 33 (NS); 100%					cohorts respectively. Seeking out shade was indicated by 45% and 50% of population in 2011 and 2012 cohorts respectively.	
Popovish, KJ (2013)[36]	United States	745 (75%); 7%	Mean age: 48	Cohort	Hospital	Lab investigations, medical records, and surveys	In HIV negative patients, temporary housing (homeless; shelter, or substance use centre) was associated with higher CA-MRSA colonization burden. In HIV infected patients, male sex, younger age, and recent incarceration were positively associated with CA-MRSA.	3
Grossberg, AL (2012)[37]	United States	PEH: 22 (63.6%); 100%<br>Housed individuals: 60 (38.3%); 0%	PEH: mean age: 47<br>Housed individuals mean age: 44	Case-control	Clinic	Medical records	Compared to housed individuals, PEH were found to have more diagnosis of malignant/pre-malignant grown; (25% vs. 6.1%, p<0.0001), however, there was no significant difference for other dermatologic conditions, such as acne, psoriasis, rosacea, dermatitis, or tinea pedis or tinea cruris. The prevalence of the skin conditions were: acne, 3.1%; psoriasis, 7.8%; rosacea, 1.6%; dermatitis (including eczema, atopic, and contact dermatitis), 12.5%; seborrheic dermatitis, 1.6%; tinea pedis, 3.1%; and tinea cruris, 1.6%.	4
Fairbairn, N (2011)[38]	Canada	428(60%); NS	Median age: 41	Cohort	Street Outreach	Interviews, medical records, and surveys	Among HIV positive PWID patients, SSTI accounted for the greatest proportion of ED visits (17%). Factors independently associated with the time for first ED visit included unstable housing and inability to receive the required healthcare services.	3
Popovich, K (2019)[39]	United States	718(100%); (46%)	Mean age: 37.6	Cohort	Jail	Lab investigations and surveys	At jail enrollment, the prevalence of MRSA colonization was 19%. Unstable housing, methamphetamine use, current/recent skin infection, and recent injection drug use were predictors of MRSA colonization.	3
Joseph, A (2019)[40]	United States	75 (100%); 100%	Age range: 18+	Cross-sectional	Shelters for PEH	Surveys	71% of participants never checked themselves for skin cancer and only 13% reported ever being screened by clinician for skin cancer. 49% of participants knew that a change in a mole's appearance and sore that does not heal was indicative of skin cancer. Black men were less likely to know that dark skin could still get skin cancer and that	3

							sunscreen should be applied 15-30 mins before sun exposure. Finally, only 21% reported using sunscreen.	
Benjaminsen , L (2018)[41]	Denmark	Shelter Users: 14,730 (79%); 100% General Population: 3,941,546(49%); NS	Mean age: 44.5	Cohort	Statistics Denmark	Statistical records	Compared to the general population, shelter users had a significantly higher morbidity due to infectious disease, lung, skin, blood and digestive diseases, injuries, and poisoning. Shelter users had statistically significant higher prevalence of skin disease among age group 23-39 (14.6% vs 4.2%; p<0.0001); and age group 40-59 (10.8% vs 4.2%; p<0.0001).	1
Dohoo, C (2019)[42]	Canada	43(100%); 100%	Mean age: 52.5	Case-control	Shelters for PEH	Interviews and medical records	Statistically significant predictors for the iGAS (either emm74 infection or colonization) were presence of previous skin condition, history of recent wounds , and younger individuals. Outbreak investigations demonstrated the need for improved wound care, infection prevention and control practices, and for early screening and detection of skin and soft tissue infections.	4
Leibler, JH (2017)[43]	United States	194(43.5%) ; 100%	NS	Cross-sectional	Boston Health care for the homeless program	Interviews and lab investigations	The prevalence of MRSA nasal colonization was 8.3%. Resistance to erythromycin (81.3%); levofloxacin (31.3%) and clindamycin (23.1%) was identified.	3
Leibler, J (2019)[44]	United States	78(64.1%); 62.8%	Mean Age: 38.7	Cohort	Hospital	Lab investigations and surveys	Prevalence of MRSA nasal colonization was 28.2% in a population of PWID. Risk factors for colonization included sleeping in a homeless shelter in the last 3 months(p= 0.02); sleeping at > 1 place during the last week (p=0.01); usage of public shower facilities in the last week (p=0.02); and sharing bedding with other people (p=0.05). No	3

							association between hand hygiene, frequency of bathing or clothes laundering, or street sleeping and MRSA colonization were observed.	
Lowrie, F (2019)[45]	Scotland	52(82.7%); 100%	Mean age: 39.8	Cohort	Drop-in clinic.	Medical records	In order to improve access, pharmacists were able to prescribe medications and wound dressings. Pharmacists were also able to diagnose a new clinical condition in 69% of patients, most commonly skin or respiratory infections.	3
Ly, TDA (2019)[46]	France	332 (96.7%);100%	Mean Age: 41	Cohort	Emergency shelter	Lab investigations, and medical records	Body lice was found in 7.4% of subjects. There was a strong association between body lice infestation and *Acinetobacter baumanni* DNA skin carriage (p=0.029). All blood culture were negative for *A. baumanni. A. baumanni* is associated with hospital acquired infections and opportunistic infections of skin, soft tissue, blood and urinary tract.	3
Dauby, N (2018)[47]	Belgium	PEH: 28 (89.3%); 100%<br><br>People not experiencing homelessness: 30 (83.3%); 0%	PEH mean age: 43.9<br><br>People not experiencing homelessness mean age: 43.5	Cohort	Hospital	Medical records	48% of adults hospitalized with a GAS infection were homeless. The incidence of GAS was 100 times higher amongst PEH. PEH had a higher proportion of GAS skin abscesses (21.4% vs. 3.3%, p=0.048), and mortality rates due to GAS infections (10% vs. 3.3%). The three deaths observed in this population were due to iGAS infections resulting in cellulitis with concomitant bacteremia and necrotizing fasciitis. PEH had lower emm-type diversity and had preferential infection with four GAS emm- subtypes types(64,77, 83, and 101) likely due to endemic circulation of these strains in this population. Preventative strategies for GAS infections in this population included wound care, and hygiene promotion.	3
Adebanjo, T (2018)[48]	United States	539 (70%); 100%	Median age: 51	Cross-sectional	Homeless facilities	Lab investigations and surveys	GAS colonization was found in 8.7% of participants. Among GAS colonized participants, GAS was cultured from 74.5% of oropoharynx and 36.2% of skin swabs. Statistically significant factors associated with GAS colonization, included younger age, sleeping outside, sharing blankets or cigarettes or clothes with others, infrequent tooth brushing, and skin breakdown on hands and arms.	3

Arnold-Reed, Diane (2018)[49]	Australia	4285(49.2 %); 100%	Mean age: 38.2	Cohort	Street-based clinic	Medical records	Psychiatric, musculoskeletal (especially skin) and respiratory conditions were most common diagnosis in this population. Indigenous patients had a significantly higher frequency of chronic skin conditions compared with non-Indigenous patients (46.1% vs. 29.7%, p <0.001).	3
Mosites, E (2018) [50]	United States	277 (70%); 100%	Mean age: 52	Cohort	Homeless facilities	Lab investigations and medical records	An outbreak of GAS colonization was identified in 9% of participants. 83% of individuals with iGAS emm26.3 infections were PEH. GAS emm26.3 strain was associated with cellulitis and necrotizing fasciitis. Mass administration of azithromycin lead to reduction in iGAS incidence from 1.5 to 0.2 cases per 1000 PEH.	3
Rizk, HI (2017)[51]	Egypt	2169 (58.6%); 100%	Median age: 9 years	Cohort	Hospital	Medical record	Skin conditions was the second most common health problem among children experiencing homelessness (16.6%). Diverse skin conditions were documented: rash (11.9%); itch(11.1%); allergic dermatitis (3.8%); scabies (3.8%); and lice infestations (3%).	3
Beijer, U (2016)[19]	Sweden	PEH: 3887 (76%);100% Control population (gender and sex matched: 11,661(NS) ;0%	Mean age for both cohorts: 43	Cohort	PEH in Stockholm city	Medical record	Compared to control population, there were higher rates of hospitalization among PEH, particularly among people age 18-35 years. The greatest difference was found in skin diseases, infections, injury / poisoning and respiratory diseases.	3
Arnaud, A (2015)[52]	France	PEH sleeping in public places: 341(93%); 100%	Mean age for PEH sleeping in public places: 44.1	Cross-sectional	PEH sleeping in public places and shelters.	Medical records and surveys	The prevalence of scabies and pediculosis corporis in individuals sleeping in public places was estimated at 6.5% and 5.4% respectively, whereas for those sleeping in shelters, it was estimated at 0.4% and 0.15% respectively. In public places, multi-variate analysis showed that being a woman, living in an abandoned place, and not possessing a sleeping bag was associated with a diagnosis of scabies. Likewise, begging, a history of pubic lice, and not taking	3

		PEH sleeping in shelters: 667(60%); 100%	Mean age for PEH sleeping in shelters: 42.8				showers in municipal baths was associated with pediculosis corporis in public places.	
Pilon, PA (2019)[53]	Canada	23(60.9%); 52%	Median age: 54	Case-control	PEH in Montreal Quebec	Lab investigation and medical records	52% of individuals infected with the iGAS emm 74 subtype were homeless, another 26% utilized homeless services but were not homeless . The most common clinical presentation was SSTI, including necrotizing fasciitis.	4
Duford, A (2019)[54]	United States	818(74%); 71%	40% of patientswere 56 years +	Cohort	Mobile health clinic in Nevada.	Medical records	The top five reasons for visits included preventive care, cognitive/ functional impairment, cardiometabolic disorders, skin issues and respiratory illness. The most frequently seen skin issues were infections, blisters, ulcerations, arthropod infestations, and wound care.	3
Ayaya, SO (2001)[55]	Kenya	191(NS); 100%	NS	Cohort	'Street children'	Medical records and surveys	Skin disease was the most common disease category among street children (50.9%). Of these cases, 26% were fungal infection; 19% were injuries; 17.2% were parasitic infections; 13.8% were acne vulgaris; 12.1% were eczema; 10.3% were viral infections; and 1.7% were genital ulcer disease.	3
Gruner, E (1994)[56]	Switzerland	117 (68%); NS	Age rage: 17-45	Case-control	Infirmary for PEH	Lab investigations and medical records	4.3% of pharyngeal swab and 17.9% of superficial wound swabs were positive for *Corynebacterium diphtheriae.* 90% of skin and pharyngeal *C. diphtheriae* infections occurred in PEH. Skin infection were described as chronic, nonhealing ulcers due to physical trauma and/or underlying dermatoses that were superinfected with *Corynebacterium diphtheriae.* Overcrowding, poor hygiene and personal proximity were associated with *Corynebacterium diphtheriae* colonization.	4
Reuler, JB (1991)[57]	United States	609 (46.8%); 100%	Mean age: 16 years and 9 months	Cohort	Drop-in Centre/clinic	Medical records	The majority of health concerns were respiratory (28.6%); followed by dermatological concerns (16.6%). Dermatologic skin conditions described were scabies, lice, wound infection, urticaria, dermatitis, and impetigo.	3

Bonilla, DL (2014)[58]	United States	203(71%); 90%	Median age: 46	Case-control	San Francisco Project Homeless connect (SFPHC)	Medical records and surveys	The prevalence of body lice and head lice was 30% and 4.9% respectively, with 3.0% of patients with both. Serologic positive *Bartonella quintana* was detected in 15.9% of patients with body lice and in 37.5% of patients with head lice. The presence of body lice was positively associated with male sex, African-American ethnicity, and sleeping outdoors, (p<0.05).	4
Ly, TDA (2017)[59]	France	2,288(95.4 %); 100%	Mean age: 43.1	Cross-sectional	Shelters for PEH	Interviews and medical records	The prevalence of body lice was 12.2%; head lice was 4.5%; and pubic lice was 3.2%. Factors associated with body lice infestations, included older age, alcohol consumption, and tobacco smoking.	4
Faccini-Martinez, AA (2017)[60]	Columbia	153 (86.3%); 100%	Mean age: 39.6	Cross-sectional	Shelter for PEH	Lab investigations and medical records	Prevalence of body louse infestation was 11.7%. The seroprevalence for Bartonella spp was found to be 19.0% and typhus group rickettsiae was found to be 56%. *Bartonella quintana* was found in 28.2% of body lice collected from individuals sampled.	3
Benkouiten, S (2014)[61]	France	125 (96%); 100%	Mean age: 56.9	RCT	Shelter for PEH	Medical records	Compared to placebo, higher prevalence of PEH receiving permethrin-impregnated underwear were free of body lice on day 14 (28% vs 9%, p=0.04); however, the difference was not sustained on day 45. On day 45, permethrin resistance was significantly higher in permethrin group compared to placebo (73% vs 45%, p<0.001).	2
Brouqui, P (1999)[79]	France	71(NS); 100%	NS	Cohort	Hospital	Lab investigations, medical records, and surveys	Positive *Bartonella quintana* serology was found in 14% of participants. Furthermore, 24% of participants had evidence of recent infection (bacteremia or seroconversion). Body lice was collected from 20% of individuals with positive *Bartonella quintna* serology. Compared to PEH without *B. quintana* bacteremia, those with bacteremia were more likely to have been exposed to lice (p=0.002); to have headaches (0.03); severe leg pain (p<0.001) and lower platelet count (p=0.006).	3
Guibal, F (2001)[62]	France	57(95); 100%	Mean age: 40	Cohort	Hospital	Lab investigations and medical records	Compared to age and gender frequency matched controls, PEH with cutaneous parasitic infections had higher prevalence of positive *Bartonella quintana* serology (54% vs. 2%, p<0.0001). Age and years of homelessness were	3

							independently associated with positive *Bartonella quintana* serology.	
Vayalumkal, JV(2009)[63]	Canada	MRSA infections cases: 81(63%); NS<br><br>MSSA infection cases: 72(56%); NS	MRSA infection mean age: 37<br><br>MSSA infection mean age: 47	Case-control	Hospital	Lab investigations and medical records.	Compared to patients with MSSA infections, patients with MRSA infections were more likely to be have experienced homelessness in the last year(p<0.001); incarceration (p<0.001); and have lived in a communal setting (p<0.001).	4
Contag, C (2017)[64]	United States	254(NS); 100%	Mean age: 48.7	Cohort	Shelters for PEH	Medical records	53.5% of medical records reviewed were associated with dermatologic disease. The top five prevalent dermatologic conditions were: (1) Inflammatory dermatoses , 22.%; (2) Superficial fungal infections, 21.0%; (3) Wounds and traumas, 16.0%; (4) Infestations, 11.7%; (5) Bacterial Infections,11.1%. Dermatologic complaints of the lower extremity were especially common. This finding may be attributed to ill-fitting shoes, difficulty performing foot and nail hygiene, spending disproportionate time sitting, standing, or walking with resulting venous stasis. This combined with common comorbidities among PEH such as neuropathy secondary to alcohol or diabetes and ischemia from arteriosclerosis predisposed them to infections and poor wound healing.	3
Shahriari, N (2017)[65]	Switzerlan d	273(53.8%) ; 100%	Mean age: 37.2	Cohort	Clinic for PEH	Medical records	The most common dermatologic conditions were acne vulgaris (18.7%), atopic dermatitis (10.6%), tinea pedis (9.2%), xerosis (8.1%), folliculitis (5.1%), and scabies (0.4%). The rate of suspected skin malignancies in this population was 2.5%. 1.8% of patients presented with lesions suspicious for melanoma, and 0.7% of patients with lesions suspicious for BCC.	3

Altshuler, J (2015)[66]	United Staets	71(NS); NS	NS	Cohort	Clinic for PEH	Interviews, medical records, and surveys	Among this population, 29% of diagnosis were inflammatory conditions; 23% were benign growth; 14% were infectious diseases; and 11% of were cancerous or precancerous lesions. Patient surveys showed that 80% of patients have not heard of BCC, and 57% reported using no sunscreen. Through education, patients showed statistically significant increase in their knowledge of BCC and sunscreen selection for sun protection.	3
Moy, J (1992)[67]	United States	NS	NS	Cohort	Hospital	NS	A survey of admissions to the inpatient dermatology ward over a 3-month period showed that 46% of patients admitted were experiencing homelessness, either living on the street or in shelters, and 48% were admitted for cellulitis or other skin infection. Furthermore, 81% of all patients admitted for cellulitis or other pyoderma were experiencing homelessness.	4
Sherman, DJ (1992)[68]	United States	214(86%); 100%	Age range: 10-21	Cohort	Clinics for PEH	Interviews, medical records, and lab investigations	Skin concerns were one of the major concerns amongst youth experiencing homelessness (31.9%).	3
Chen, B (2012)[69]	United States	299 (92%); 100%	NS; 62% were between 36-55 years of age.	Cohort	Shelter for PEH	Medical records and surveys	Dermatologic foot concerns included onychomycosis (30%); foot injuries (27%); calluses (26%); corns (19%); athlete's foot (24%); ingrown nails (15%); immersion foot (5%); ulcers (4%); warts (4%); peripheral artery disease (3%); and frostbite (2%).	3
Mosites, E (2019)[70]	United States	PEH: 970(NS) ; 100%<br><br>Control:288921(NS);0%	Mean age of PEH with GAS: 51<br><br>Mean age of controls	Census/Cross sectional study	PEH in Anchorage, Alaska	Medical and statistical records	Compared to the general population, PEH were 53.3 times more likely to have iGAS infection. iGAS infections among PEH were associated with being male and alcohol-use disorder. PEH with iGAS infection were more likely to have a diagnosis of cellulitis or necrotizing fasciitis. Amongst PEH, the most common iGAS emm subtypes were 91, 82 and 49, whereas emm 1, 49, 82, and 89 were more common in general population.	1

			with GAS: 54					
Lowe, CF (2011)[71]	Canada	33(60.1); NS	Mean age: 41.7	Cohort	VDES	Medical records	*Cornyebacterium diptheriae* is endemic in Vancouver's urban core. All *Cornyebacterium diptheriae* isolates were nontoxigenic with strains of multilocus sequence76 predominating. The main choice treatment was penicillin G.	3
Romney, MG (2006)[72]	Canada	7 (71%); NS	Mean age: 46.9	Cohort	VDES	Medical records	Skin colonization/infection with *Corynebacterium diphtheriae,* homelessness, injection drug use, and diabetes mellitus were factors associated with *C. diphtheriae* bacteremia.	4
Foucault, C (2006)[73]	France	33(NS);100 %	NS	Cohort	Shelter for PEH	Medical records	Over the 14 day ivermectin treatment period, the prevalence of lice infested individuals fell from 84.9% to 18.5%. Although this effect was not sustained at day 45 (perhaps due to re-infestation), it establishes that ivermectin plays a novel role in control of body louse infestations in humans. On day 14, lice was not completely eradicated due to re-infestation, possible alcohol related poor absorption of the drug, and possible under dosing of ivermectin.	3
Truong, A (2019)[74]	United States	507(68.8%) ; 100%	Age at first visit: 48.9	Cohort	Clinic dedicated to PEH	Medical records	The prevalence of dermatologic diagnosis categories amongst this population was: benign conditions (42.6%); dermatitis/psoriasis (26.6%); infection(21.7%); premalignant/malignant(20.5%); acne/rosacea (7.5%); and others (28.2%). Infectious and pre-malignant/malignant skin diagnosis were more commonly diagnosed in men, whereas acne/rosacea were more commonly diagnosed in women. Statistically significant factors associated with follow-up adherence amongst PEH included older age, male sex, more skin diagnosis, premalignant/malignant diagnosis, in clinic procedures, shorter recommended follow-up interval and fewer mental health diagnosis.	3
Truong, A (2020) [76]	United States	129(67%); 100%	Median age: 49	Cohort	Clinic dedicated to PEH	Surveys and medical records	Dermatologic conditions included "rash"(44%, most commonly dermatitis); actinic keratosis/skin cancer (15%); and infections (11%, most commonly folliculitis). The average DLQI for acne, psoriasis, eczema and skin cancer were generally higher amongst PEH compared to scores	3

							from non-homeless populations. High DLQI scores corresponded to lower HRQL scores and were associated with non-white patients, poorer skin health, rash or infectious diagnosis. A significant majority of participants experienced difficulties affording skincare products.	
Truong, A (2020) [75]	United States	PEH: 174(NS); 100%<br><br>Non-homeless patients: 849(NS); 0%	NS	Case-control	Clinic dedicated to PEH	Medical records	Treatment disparities for common skin conditions among PEH compared to the non-homeless population demonstrated a pattern of less diagnostic inquiry, less aggressive intervention and fewer recommendation for follow-up for common skin conditions, despite the same dermatologists treating the same groups. Potential explanation for the differing behaviour among the dermatologists may include lack of knowledge of available resources at the homeless clinic, implicit biases, concern for patient adherence or resource stewardship, or tendency to overmedicalize non-homeless patients.	4
Harris, M (2020)[77]	United Kingdom	455(75%); 78%	Mean age: 46	Qualitative study	Specialist drug services, homeless hustles, and daycare centres	Interviews and surveys	Survey data and interviews demonstrated that engagement with medical system was a last resort with admission to hospital in critical or near death conditions.	3
Valenciano, SJ (2020)[78]	United States	Neither PEH or PWID: 10,807(54%); 0%<br><br>PEH: 531(77%); 100%	Mean age of patients who were neither PEH or PWID: 53	Cohort	PEH in 10 US cities	Medical and statistical records	Patients with a history of IDU or homelessness accounted for 21% of iGAS infection cases. iGAS infections were 80 times greater among PEH compared to the general population. Higher prevalence of skin breakdown amongst PEH may suggest that skin breakdown was a prominent route for acquiring iGAS infections.	3

			Mean age of PEH: 51					
Van Laere, I (2001)[15]	Netherlands	364 (84%);100 %	Mean age: 43	Cross-sectional	Shelter for PEH	Medical records	In the first consultation, skin problems were the most prevalent concern (26%). Dermatologic concerns included traumatic injuries, infected wounds, tramp's feet, scabies, lice, abscess, cellulites, and erysipelas.	3

Abbreviations: BCC, Basal cell carcinoma; CA-MRSA, community acquired-Methicillin Resistant *Staphylococcus Aureus*; CIRI, Cutaneous injection related infection; DLQI, Dermatology quality of life index; ED, Emergency Department; HRQL, Health related quality of life; HIV, Human Immunodeficiency virus; IDU, injection drug use; iGAS, invasive *Group A Streptococcus;* MRSA, Methicillin Resistant *Staphylococcus Aureus;* MSSA, Methicillin Susceptible *Staphylococcus Aureus;* NS, Not Specified in article; PEH, persons experiencing homelessness; PWID, persons who inject drugs; RCT, Randomized controlled trial; SCC, squamous cell carcinoma; SIF, safe injection facility; SSTI, skin and soft tissue infections; STI, sexually transmitted infections; US, United States; VDES, Vancouver Downtown East Side.

Table S2. Summary of case reports included in our scoping review, including information on demographics, past medical history, clinical findings, diagnosis, outcome, and significance of case study.

Author Last Name (Year)	Country	Gender; Age	Past Medical History	Clinical Findings	Diagnosis	Outcome	Significance
Wrenn, K (1991) [81]	United States	M; 38	Tobacco use; alcohol use disorder with a history of pancreatitis.	Afebrile; bilateral swelling and patchy erythema to just above malleoli; violaceous discoloration of the plantar surfaces of both feet and scaling in the interdigital spaces; tenderness of both soles; decreased pin and vibration sensation.	Immersion Foot	Full recovery within 48 hours of bed rest, drying of feet, and ibuprofen administration.	Higher prevalence of immersion foot amongst PEH.
		M; 34	Cocaine use	Bilateral painful edema of feet; tenderness over the soles with wrinkling; pitted keratolysis; scaling and mild erythema.	Immersion Foot	Significant improvement within 24 hours after air dying, foot elevation, ibuprofen and cephalexin administration.	
Uhoda, E (2004) [82]	Belgium	3 men experiencing homelessness:<br>Patient 1: M; 27<br>Patient 2: M; 28<br>Patient 3: M; 32	Alcohol-related liver dysfunction;<br>malnutrition including vitamin A and carotene deficiency	Hyperkeratinisation of epidermis and lining of pilosebaceous follicles; xerosis; extensive epidermal melanisation.	Vitamin A deficiency	Only patient 2 accepted treatment. After 10 weeks of following better dietary habits and Vitamin A supplementation, the follicular keratosis resolved.	Early detection of vitamin A deficiency may prevent its complications, such as keratomalacia corresponding to softening and perforation of cornea, which may ultimately lead to blindness. In this care report, early recognition of Vitamin A deficiency through ULEV (

							ultraviolet light enhanced visualization) was considered a useful tool for screening at risk populations.
Savvidou, S (2014)[83]	Greece	M; 50	"Patient was unreliable in providing past medical history".	Confusion; disorientation; cachexia; dehydration; extensive brown pigmentation and scaling eruptions on a red base along the dorsa of his hands and sun-exposed surface of his arms. No abnormalities in cranial or tendon reflexes.	Pellagra	Intravenous fluids, high caloric nutrition administration, and nicotinamide 300 mg orally improved patient's skin lesions.	Clinicians should keep a high index of suspicion for pellagra in at risk patients, including PEH, those with alcohol use disorder, and those with end-stage malignancy or HIV.
Lenardis, MA (2014)[84]	Canada	M: 50	Seizure	A 6x6 mm nevus on chest since childhood; slightly raised with irregular pigmentation; no pain or itching. It has been growing and becoming more pigmented recently.	Benign Nevus	Tele-dermatology was employed; images were taken of nevus and sent for consultation to a dermatologist. No treatment was required	Tele-dermatology may be a suitable management option for PEH with no fixed address, and therefore cannot be contacted for the referral or follow-up. Tele-dermatology accommodated the patient's lack of accessibility while still maintaining the standard of care.
Martins, LG (2014)[85]	Brazil	M; 47	NS	Dermatologic exam revealed presence of excoriated lesions at the occipital and cervical regions, as well as lice and several white spots adhered to beard hairs; hyperchromic lesions on the lower limbs.	Body lice infestation by *Pediculus humanus corporis*	Oral Ivermectin and shaving of body and head hair.	Oral Ivermectin is a suitable treatment option for individuals with extensive body lice infestation.
Kertesz, S (2001)[86]	United States	M; 58	Tobacco usage; bipolar disorder	Chest Pain; fatigue; night sweats; fever; weight loss; burning sensation in the mouth; nausea; vomiting; diarrhea; cutaneous burning sensations; bronze-colored skin-pigmentation; cough; pronounced emotional	Pellagra	Regular meal provision at shelter; niacin 100 mg PO BID resolved his presenting symptoms in 2 weeks.	Clinicians should keep a high index of suspicion for pellagra in at risk patients, including PEH, those with alcohol use disorder, and those with end-stage malignancy or HIV.

				liability for the last few month.			
		M; 55	Allergic Contact dermatitis with cellulitis.	Extensive cutaneous weeping, edema, erythema, fissuring, and scale over the ears, face, collar area, and forearms.		A prescription of multi-vitamins, niacin 100 mg PO daily and triamcinolone cream helped resolve the patient's rash within few weeks.	
Singh, P (2015)[87]	India	F; 35	Schizophrenia	Itchy, crusted, and scaly lesions on chest, extremities and trunk.	Dermatosis neglecta	Cutaneous debris and crusting was cleaned with soap water followed by alcohol swab cleaning.	Dermatosis neglecta is a chronic, progressive dermatologic disorder resulting from accumulation of sebum, keratin, dirt and other epidermal debris forming a hyper-pigmented, scaly, plaque like lesion. It occurs frequently on neglected skin areas due to improper cleaning or scrubbing.
Monsuez, J (1995)[88]	France	M; 62	Hypertension; alcohol use disorder	Non-healing leg ulcer for 4 months. The ulceration appeared after a minor skin wound, and it grew gradually to reach 7 cm in diameter, and 5mm in depth. It was covered with a purulent membrane and surrounded by necrotic zone.	Cutaneous diphtheria	Wound Care and IV penicillin resulted in rapid improvement of the ulcer within 3 weeks.	Cutaneous diphtheria may be suspected in impoverished individuals with poor hygiene and without adequate immunizations.
Guss, DA (2011) [89]	United States	M; 61	Cellulitis, heavy louse infestation	Light-headedness; generalized weakness; skin did not reveal any discoloration or rash, and was remarkable for numerous live lice on the body and scalp, with louse nits in the hair.	Severe anemia due to louse infestations (Hemoglobin less than 6 gm/dL)	Permethrin 5% lotion and blood transfusion.	Clinicians should be suspicious of anemia due to severe and prolonged infestation.

		M; 55	Excessive alcohol consumption; heavy louse infestation	Dyspnea			
		M; 43		Rash			
		M; 52		Weakness			
		M; 50		Assault			
		F; 54		Abdominal pain			

Abbreviations: BID, twice a day; F, Female; gm/dL, grams per deciliter; IV, intravenous; M, Male; mg, milligram; NS, Not Specified in article; PO, orally.

Figure S1: PRISMA Study Selection Flow Diagram

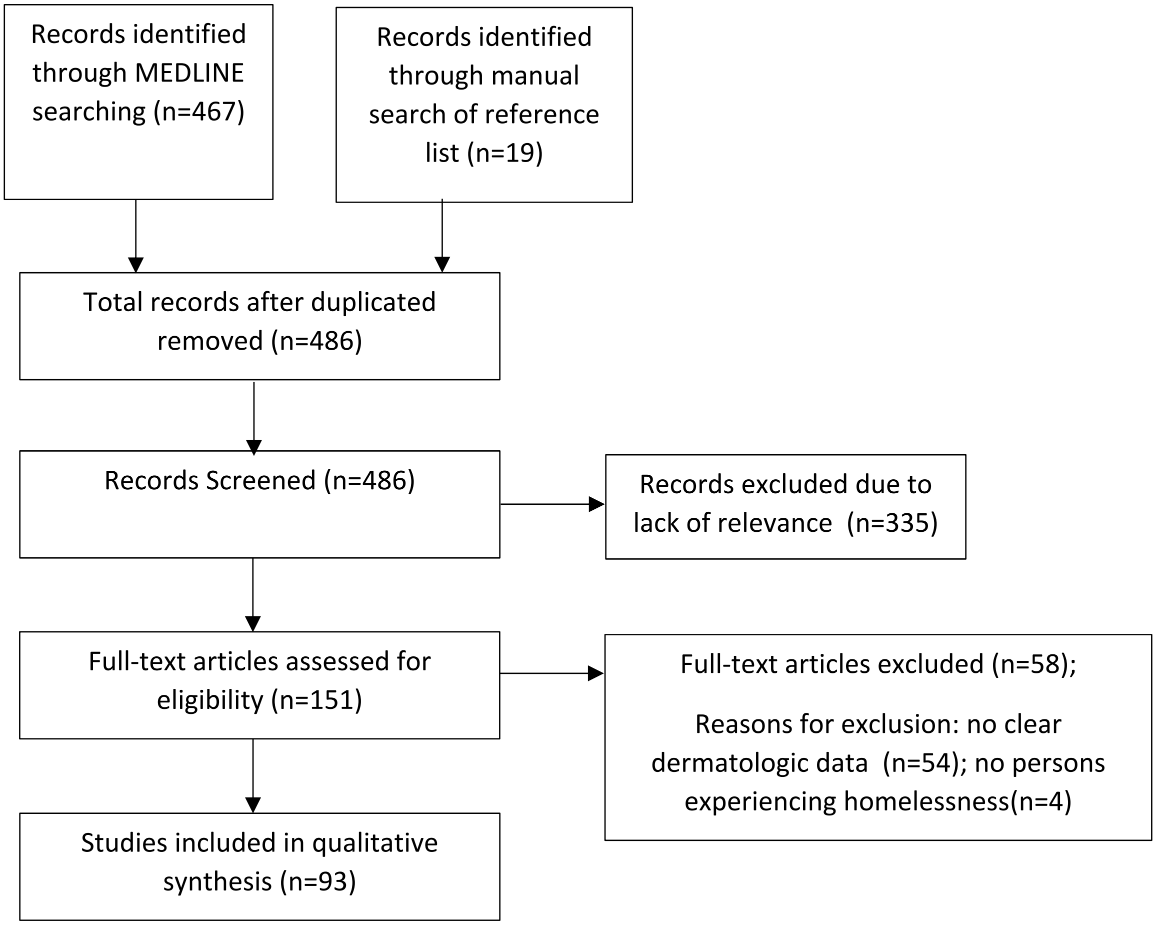

Supplement: Supplementary Material 1 - Supplemental material for Understanding Dermatologic Concerns Among Persons Experiencing Homelessness: A Scoping Review and Discussion for Improved Delivery of Care [file sj-pdf-1-cms-10.1177_12034754211004558.pdf]
